# Adverse effects of the ‘Noakes’ diet on dyslipidaemia

**Published:** 2014

**Authors:** C Schamroth

**Affiliations:** Milpark Hospital, Johannesburg

## Dear Sir

There has been much hype and controversy over the so-called ‘Noakes’ diet. This diet advocates a low-cardohydrate, high-fat and high-protein intake. As previously reported in the Journal, Noakes has expressed the view that this diet coupled with exercise could have a favourable impact on lipid levels and potentially avoid the need for drug therapy.^1^ In that same report, it was noted that this has not been subjected to scientific validation. More recently the ability of the Noakes diet to give better weight-control results than a ‘balanced diet’ has been questioned.^2^

I have recently seen a patient who had been diagnosed with dyslipidaemia several years ago and was receiving rosuvastatin 5 mg daily. A lipogram taken in April 2012 (on therapy) showed a total cholesterol (TC) level of 5.1 mmol/l and low-density lipoprotein cholesterol (LDL-C) of 2.9 mmol/l, respectively. After two months on the Noakes diet, she had the lipogram repeated. This now showed a TC of 12.9 mmol/l and LDL-C of 9.6 mmol/l, respectively. This was in the absence of diabetes mellitus and hypothyroidism.

While one case does not make a scientific study, it should nevertheless alert practitioners involved in treating patients with dyslipidaemia and attempting to lower cardiovascular risk, that subjects on the Noakes diet should be cautioned as to the potential risks and have their lipid profiles closely monitored.
